# Will a lack of fabric durability be their downfall? Impact of textile durability on the efficacy of three types of dual-active-ingredient long-lasting insecticidal nets: a secondary analysis on malaria prevalence and incidence from a cluster-randomized trial in north-west Tanzania

**DOI:** 10.1186/s12936-024-05020-y

**Published:** 2024-06-28

**Authors:** Eliud Andrea Lukole, Jackie Cook, Jacklin F. Mosha, Elizabeth Mallya, Tatu Aziz, Manisha A. Kulkarni, Nancy S. Matowo, Jacklin Martin, Mark Rowland, Immo Kleinschmidt, Alphaxard Manjurano, Franklin W. Mosha, Natacha Protopopoff

**Affiliations:** 1 Department of Parasitology, National Institute for Medical Research, Mwanza Medical Research Centre, Mwanza, Tanzania; 2 Department of Infectious Disease Epidemiology, MRC International Statistics and Epidemiology Group, London School of Hygiene and Tropical Medicine, London, UK; 3 Wits Research Institute for Malaria, School of Pathology, Faculty of Health Sciences, University of the Witwatersrand, Johannesburg, South Africa; 4 Southern African Development Community Malaria Elimination Eight Secretariat, Windhoek, Namibia; 5 Kilimanjaro Christian Medical University College, Moshi, Tanzania; 6 School of Epidemiology and Public Health, University of Ottawa, Ottawa, ON Canada; 7 Department of Disease Control, London School of Hygiene and Tropical Medicine, London, UK

## Abstract

**Background:**

The Dual-Active Ingredient long-lasting insecticidal nets (Dual-AI LLIN) have been developed to counteract the reduced efficacy of pyrethroid (PY)-only nets due to widespread pyrethroid insecticide resistance in malaria vector mosquitoes. They constitute half of the nets distributed in sub-Saharan Africa between 2022 and 2024. However, their effectiveness once they develop holes is unclear, particularly in pyrethroid-resistant settings. This study evaluates the textile integrity of three dual- AI LLINs compared to standard PY LLN, over 3 years of use in a community in Tanzania and the associated impact on malaria prevalence and incidence.

**Methods:**

A secondary analysis of data from a randomized controlled trial (RCT) in North-western Tanzania was conducted to evaluate the effectiveness of α-cypermethrin only; pyriproxyfen and α-cypermethrin (PPF-PY); chlorfenapyr and α-cypermethrin (chlorfenapyr-PY); and the synergist piperonyl butoxide and permethrin (PBO-PY) LLINs on malaria infection prevalence and case incidence. The association between the net textile condition and 1/malaria prevalence over 3 years of use between 2019 and 2022, and 2/malaria case incidence in a cohort of children over 2 years of follow-up was assessed between 2019 and 2021.

**Results:**

There was no significant association between damaged (OR 0.98, 95% CI 0.71–1.37, p-value  = 0.655) and too-torn (OR 1.07, 95% CI 0.77–1.47, p-value = 0.694) compared to intact nets on malaria prevalence for all net types. However, there were reduced rates of malaria case incidence in children sleeping under a net in good condition compared to too-torn nets (incidence rate ratio (IRR) 0.76 [95% CI 0.63–0.92], p = 0.005). Malaria incidence was also consistently lower in too-torn PBO-PY LLIN (IRR = 0.37 [95% CI 0.19–0.72], p = 0.003) and chlorfenapyr-PY LLIN (IRR = 0.45 [95% CI 0.33–0.97], p = 0.053) compared to an intact PY-only LLIN during the first year of follow up. In year 2, the incidence was only significantly lower in intact chlorfenapyr-PY LLIN (IRR = 0.49 [95% CI 0.29–0.81], p = 0.006) compared to intact PY LLIN.

**Conclusion:**

The study confirmed that sleeping under a chlorfenapyr-PY LLIN or PBO-PY LLIN offered superior protection to pyrethroid-only nets even when torn. Preventing the development of holes is essential as they impact the level of protection offered against malaria infection.

*Trial registration*: ClinicalTrials.gov, number (NCT03554616)

**Supplementary Information:**

The online version contains supplementary material available at 10.1186/s12936-024-05020-y.

## Background

Malaria prevention has relied on mosquito nets treated with pyrethroid insecticides for decades [1]. Scaling up of long-lasting insecticidal nets (LLINs) has averted an estimated 2 billion cases and 12 million deaths between 2000 and 2021. Due to the emergence and spread of pyrethroid insecticide resistance in malaria vector mosquitoes, new classes of LLINs combining a pyrethroid and a second insecticide with a different mode of action [2, 3], or a pyrethroid and synergist piperonyl butoxide (PBO) [4] have been developed as alternatives to pyrethroid only LLINs [5]. The addition of these classes of LLINs to the market of malaria vector control products is vital to help mitigate further development of insecticide resistance [1]. Several randomized controlled trials (RCT) have shown the superior efficacy of the combination of pyrethroid and PBO (trade names: Olyset Plus and Permanet 3) [6, 7], pyrethroid- chlorfenapyr insecticides (Interceptor G2) [8, 9], and some nets treated with pyrethroid-pyriproxyfen insecticides (Olyset Duo [10]), compared to standard pyrethroid-only LLIN.

Based on the evidence generated by these RCTs, the new net classes are now being rolled out on a large scale. In sub-Saharan Africa, around 350 million have already been distributed since 2018, and half of the nets distributed between 2022 and 2024 were PBO-pyrethroid nets or dual active ingredient nets, with these numbers set to increase [11] as these nets are gradually replacing pyrethroid-only nets in areas of pyrethroid resistance. However, the longer-term effectiveness of a net is impacted by its functional survival in field conditions [12]. Several studies have reported reduced effectiveness of holed LLINs in areas with pyrethroid resistance; however, these studies tend to be laboratory-based [13], experimental-hut trials [14–16], and assessing pyrethroid-only treated LLINs [17–19].

The present study reports on a secondary analysis of a large randomized controlled trial in Tanzania that evaluated the effectiveness of dual-AI LLINs. In the first report, it was showed that combining pyrethroids with either chlorfenapyr or PBO provides further protection against malaria infection prevalence, malaria incidence, and entomological indices over 1 or 2 years of use compared to standard LLIN [8]. This paper presents the textile integrity of three dual-AI LLINs; chlorfenapyr-PY LLIN, pyriproxyfen-PY LLIN, and PBO-PY LLIN, over time and examine the relationship of net fabric quality with malaria prevalence and incidence.

## Methods

### Study design and settings

The study took place in 17 wards (72 villages) on the southern border of Lake Victoria, Misungwi district (latitude 2°51′00.0"S, longitude 33°04′60.0"E), Mwanza region, in north-western Tanzania. This is a secondary analysis of a four-arm, double-blinded cluster randomized trial (CRT) that assessed the effectiveness of dual-AI LLINs on malaria outcomes [8, 20, 21]. The following treatments were randomly allocated to 21 clusters each (Additional file 1): Interceptor (alpha-cypermethrin, [control] arm), Interceptor G2 (chlorfenapyr-PY LLIN (alpha-cypermethrin + chlorfenapyr), Royal Guard (Pyriproxyfen-PY LLIN (alpha-cypermethrin + pyriproxyfen), and Olyset Plus [permethrin + piperonyl butoxide (PBO)]. In September/October 2021 (33 months post net distribution), the Tanzanian National Malaria Control Programme (NMCP) distributed 40,000 Olyset Plus in the study area via the school net programme (SNP).

### Participants

Malaria infection prevalence was measured during repeated cross-sectional surveys at 12 months (t12; January/February 2020), 18 months (t18; July/August 2020), 24 months (t24; January/February 2021), 30 months (t30; July/August 2021), and 36 months (t36; January/February 2022) post-intervention. Two children aged between 6 months and 14 years from each of 45 randomly selected households per cluster were tested for malaria infection using rapid diagnostic tests (RDT) (CareStart malaria HRP2 [pf], DiaSys, Wokingham, UK). In each cluster, the textile integrity of study LLINs was assessed in at least 13 randomly selected houses (out of 45) at t12, t24, and t30 and in at least 16 households (to account for fewer nets remaining in the households) at the t36 months survey. Due to the COVID-19 pandemic, holes in the nets were not assessed at t18 to adhere to safety protocols. This secondary analysis is restricted to children in households selected for net textile integrity assessment.

A cohort of 2940 children (35 children per cluster on average) aged 6 months to 10 years was recruited after net distribution in February 2019 and followed for one year until January 2020 to assess malaria case incidence. A second cohort of 3360 (40 children per cluster) was recruited 1 year after net distribution in February 2020 and followed up for 1 year until January 2021.

### Procedures

Between January 26th and 28th, 2019, the four types of nets were distributed to study arms as allocated and detailed elsewhere [8]. In each household selected for fabric integrity, a maximum of 3 study LLINs used the previous night were randomly selected for hole assessment, with priority given to the net used by the selected children for malaria infection prevalence testing.

In the cohort, malaria parasitaemia was measured bi-weekly at a central meeting point. Children with fever (tympanic temperature ≥ 37.5 °C) or a history of fever in the past 48 h were tested for malaria parasites by rapid diagnostic test (CareStart malaria HRP2/pLDH [pf/pan] combo, DiaSys, Wokingham, UK). Children with a positive rapid diagnostic test or minor illness were treated by trained study nurses/clinicians as per national guidelines.

At the end of each cohort year (last follow-up visit) from 12th December 2019 to 28th January 2020 for year 1, and from 1st December 2020 to 27th January 2021 for year 2, guardians/child caretakers of all cohort children were asked to bring the nets that a cohort child had been using for textile integrity assessment by trained field-workers. During these last cohort visits, alongside the net integrity assessment, malaria parasitaemia was measured in all children regardless of whether they had a fever or a history of fever. These data provide a direct link between the net condition and malaria infection status of the net’s user since the cohort child slept under the same net throughout the previous year. Every 3 months, the community health workers (CHWs) visited the cohort children to monitor and record net usage, and to monitor that the appropriate net was in use. These nets were labelled with the child names at the beginning of the cohort.

The number and size of holes, hole location on the net, and type of holes were recorded for each selected study net. The size was classified into four categories per WHO guidelines: size 1 = 0.5–1.99 cm, size 2 = 2–9.99 cm, size 3 = 10–25 cm, and size 4 =  > 25 cm. The size of the holes was estimated by superimposing transparent plastic with illustrations of hole sizes. The hole surface area (HSA) for each net was then calculated as the number of holes counted multiplied by the estimated hole areas as per World Health Organization (WHO) guidelines [22] as follows: HSA = (1.23 × no. of size1 holes) + (28.28 × no. of size2 holes) + (240.56 × no. of size3 holes) + (706.95 × no. size4 holes). Based on the HSA, each net was then categorized as good (HSA: ≤ 79 cm^2^), damaged (HSA: 80–789 cm^2^), or too-torn (HSA: > 789 cm^2^). The HSA was used instead of the standard practice of using proportionate Hole Index (pHI) metric that uniformly divides the hole surface area by 1.23 to obtain the weights (1, 23, 196, and 576) due to the following reasons: 1/HSA is readily quantifiable and easily understandable to readers as it uses units (square centimetres) compared to unitless pHI. 2/Both pHI and HSA allocate same number of nets in the recommended categories: good, damaged and too-torn and, therefore, cannot affect the analysis conducted. Hole types included: holes at the hanging points, holes caused by tears, holes caused by burns, holes caused by rodents, and holes caused by sharp objects.

Data collection was done on smartphones using the Open-Data-Kit (ODK) software. Data from each field team was directly uploaded to a secure database at the London School of Hygiene and Tropical Medicine (LSHTM). After completion of the surveys, datasets were transferred to STATA release 15 (StataCorp, College Station, TX, USA) for further aggregation, cleaning, and preparation for analysis.

### Outcomes

The study outcomes were malaria prevalence in children aged 6 months -14 years and incidence in children aged 6 months -10 years using dual-AI LLINs compared with standard PY LLIN with different textile conditions.

### Statistical analysis

The analysis for this study was restricted to study nets distributed in January 2019. The cross-sectional data collected at t30 were excluded from analysis due to seasonality (collected during the dry season while the rest of the surveys were in the rainy season).

Household social and economic wealth indices were constructed and analysed by Principal component analysis (PCA) and were subdivided into wealth tertiles. HSA values log-transformed to normalize the distribution. Wald tests of multiple comparisons of means were used to compare hole surface area differences between study LLINs. For cross-sectional data, the association between net physical condition and malaria infection prevalence was assessed using mixed-effects logistic regression. The association between net physical condition and cumulative incidence of malaria infection in cohort study was analysed by mixed-effects Poisson regression with individual follow-up time specified as an offset and cluster set as a random effect. Furthermore, each dual-AI LLIN in different physical condition were compared to good PY LLIN on malaria infection to assess their superiority in protection. Interactions between net type and net physical condition, survey timepoint and net type were examined.

## Results

**Figure1:**
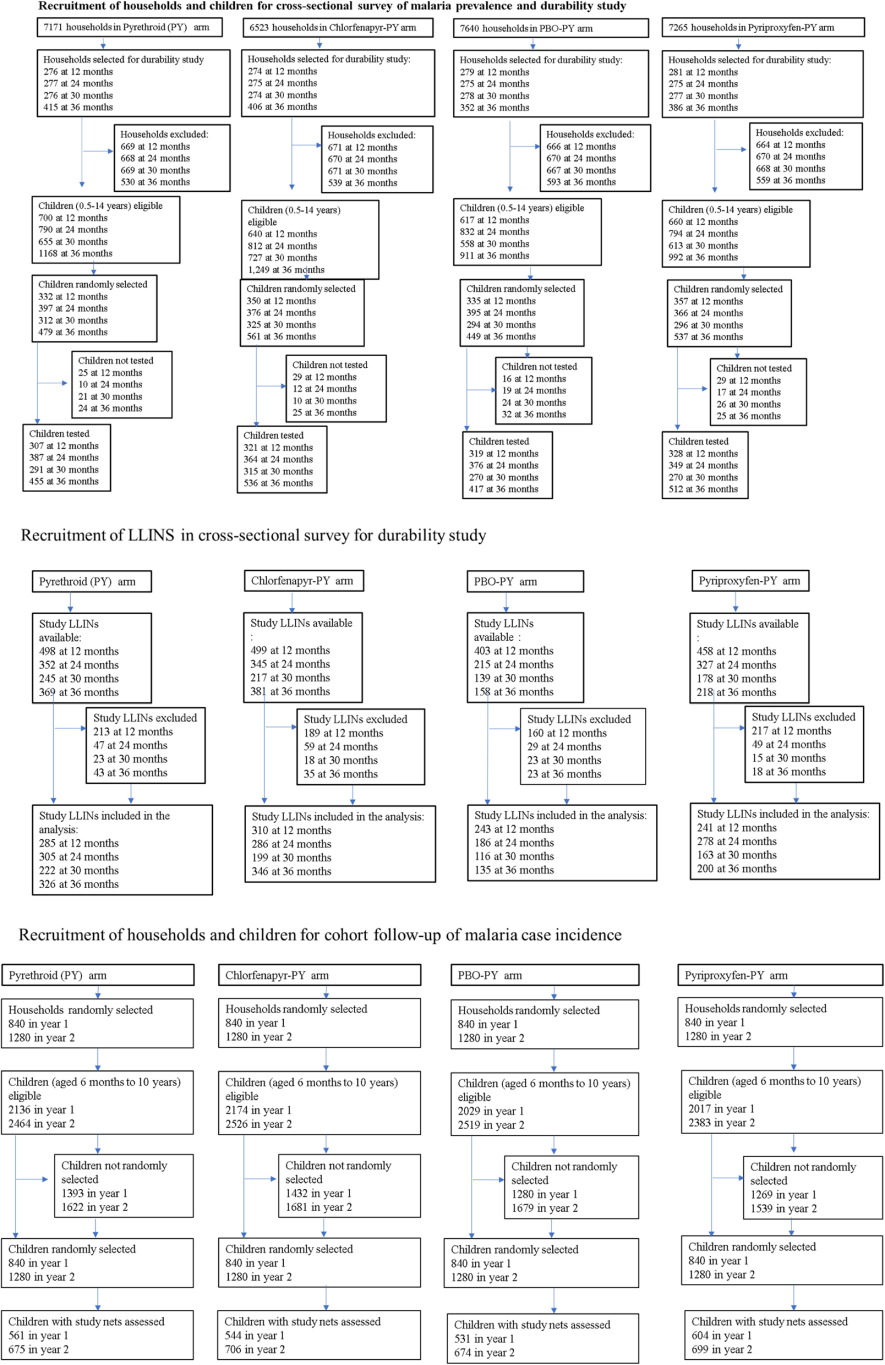
Trial profile

A total of 4876 households were selected for textile durability assessment over the four cross-sectional surveys between 07th January 2020 and 10th February 2022. Of these, 67.4% (n = 3284) consented to participate in the study. The reasons for non-consents included: 12.6% (n = 612) no children of eligible age; 9.9% (n = 485) dwellings vacant; 6.6% (n = 323) dwelling not found; and 3.5% (172) refused to participate. From the consenting households, 5817 children were tested for malaria infection, and 5060 study LLINs (1464 PY-LLIN, 1500 chlorfenapyr-PY LLIN, 1181 pyriproxyfen-PY LLIN, and 915 PBO-PY LLIN) were assessed for fabric integrity (Fig. 1). A total of 1146 children surveys at t30 were not included in the malaria prevalence analysis as they were surveyed in different season (dry). At baseline (October 2018), malaria prevalence measured in children aged 6 months to 14 years was 44% (1948/4403), balanced across study arms [20].

Ownership of any net (at least 1 net per household) remained high over the study period, from 99.7% 3 months after net distribution to 96.2% (1036/1077) at t36. Ownership of study net (≥ 1 study LLIN per house) declined over the 3 years of the study from a high of 92% (697/755 houses) after t12 in January 2020 to 62% (672/1077) in January 2022 (Table 1). Reported study net use recorded during cross-sectional surveys also declined over the 3 years of the study from 72% (3155/4373) three months after the mass distribution in January 2019 [8] to 23% (2068/9044) in January 2022 at t36 (Table 1). The lowest study net use was in the PBO-PY group at the t36 timepoint [19% (286/1469)]. Between t30 and t36, the ownership of other PBO-PY LLIN in the study area (in all arms) increased from 13% (229/1723) to 33% (1076/3263) due to the local government-led top-up campaigns through the School Net programme (SNP) (Additional file 6).

**Table 1:** Household, socioeconomic, and net characteristics of participating households in the cross-sectional surveys

Covariates	Cross-sectional survey			
Cross-sectional survey timepoint	12 months	24 months	30 months	36 months
Mean number of people per household (sd), N	7.1 (3.0), 755	8.1 (3.4), 803	8.0 (3.6), 649	8.4 (3.8), 1077
Mean number of children (< 15 years) per household (sd), N *	3.6 (1.9), 755	4.2 (2.1), 803	4.0 (2.2), 649	4.2 (2.2), 1077
Mean number of sleeping spaces used last night (sd), N	2.9 (1.4), 755	3.2 (1.6), 803	3.2 (1.5), 649	3.5 (1.7), 1077
Households characteristics				
% electricity (95% CI), N	24.5 (21.6–27.7)	27.3 (24.3–30.5)	36.1 (32.5–39.8)	37.7 (34.9–40.6)
% open eaves: (95% CI), N	32.1 (28.8–35.5)	34.1 (30.9–37.5)	34.9 (31.4–38.7)	31.57 (28.9–34.4)
Main housing materials				
% floor: earth/sand (95% CI), N	64.8 (61.3–68.1)	67.6 (64.3–70.8)	58.6 (54.7–62.3)	61.8 (58.8–64.6)
% roof: tin (95% CI), N	71.9 (68.6–75.0)	72.7 (69.5–75.7)	77.2 (73.8–80.3)	74.5 (71.8–77.0)
% walls: unburnt bricks or mud (95% CI), N	79.3 (76.3–82.1)	78.5 (75.5–81.2)	76.4 (73.0–79.5)	76.9 (74.3–79.3)
% no ceiling (95% CI), N	97.5 (96.1–98.4)	97.9 (96.6–98.7)	96.5 (94.7–97.6)	96.8 (95.5–97.7)
% plastered walls	67.3 (63.9–70.5)	63.0 (59.6–66.3)	67.5 (63.8–71.0)	67.3 (64.5–70.1)
Mean number of mosquito nets owned per household (sd), N				
Any LLIN	3.8 (1.8), 755	3.1 (1.6), 803	2.7 (1.6), 649	3.0 (1.8), 1077
Study LLIN	2.5 (1.5), 755	1.6 (1.2), 803	1.2 (1.1), 649	1.1 (1.1), 1077
Mean number of nets used last night per household (sd), N				
Any LLIN	2.4 (1.3), 755	2.4 (1.3), 803	2.0 (1.3), 649	2.3 (1.5), 1077
Study LLIN	1.6 (1.2), 755	1.2 (1.1), 803	0.9 (1.0), 649	0.8 (0.9), 1077
Proportion of households with at least 1 net % (n/N)				
Any LLIN	100 (755/755)	98.5 (791/803)	95.5 (620/649)	96.2 (1036/1077)
Study LLIN	92.3 (697/755)	78.3 (629/803)	66.6 (432/649)	62.4 (672/1077)
Proportion of participants reporting using a net the night before % (n/N)				
Any LLIN	81.3 (4306/5294)	75.4 (4899/6502)	62.3 (3220/5170)	67.8 (6128/9041)
Study LLIN	51.6 (2805/5435)	37.4 (2432/6502)	27.8 (1439/5171)	22.9 (2068/9044)
Varieties of nets available in the household	N = 3010	N = 2513	N = 1723	N = 3263
% Pyrethroid (PY) LLIN (n)	16.5 (498)	14.0 (352)	14.2 (245)	11.3 (369)
% Chlorfenapyr-PY LLIN (n)	18.5 (557)	13.7 (345)	12.6 (217)	11.7 (318)
% PBO-PY LLIN (n)	13.4 (403)	8.6 (215)	8.1 (139)	4.8 (158)
% Pyriproxyfen-PY LLIN (n)	15.2 (458)	13.0 (327)	10.3 (178)	6.7 (218)
% Permanet 2.0 (n)	17.4 (525)	19.2 (482)	20.3 (349)	16.6 (543)
% Olyset net (n)	13.9 (419)	23.8 (597)	18.6 (312)	13.7 (447)
% Olyset Plus (distributed by NMCP) (n)	0.3 (9)	6.37 (160)	13.3 (229)	33.0 (1076)
% Others nets (n)	1.3 (141)	0.6 (35)	1.2 (45)	1.0 (71)
Number of children tested for malaria (n/N)	1275/1374	1476/1534	1146/1227	1920/2026
% Malaria infection in 6 months-14 years children (95% CI)	18.7 (16.6–20.9)	38.4 (35.9–40.9)	45.6 (42.7–48.5)	31.1 (29.1–33.2)

*SD: *standard deviation; *CI*: confidence intervals; *N*: total number of observations, *NMCP*: National Malaria Control Programme

In the nets collected during cross-sectional surveys, overall, at t12, 54% (597/1110) of the study nets had at least one hole of any size (53% (152/285)-pyrethroid (PY) LLIN, 48% (162/341)-Chlorfenapyr-PY LLIN, 52% (125/241)-Pyriproxyfen-PY LLIN, and 65% (158/243)-PBO-PY LLIN), and this increased to 82% (830/1007) (84% (274/326)-PY-LLIN, 81% (280/346)-Chlorfenapyr-PY LLIN, 81% (162/200)-Pyriproxyfen-PY LLIN and 84% (114/135)-PBO-PY LLIN) at t36. Similarly, the mean Hole Surface Area (HSA) increased from 340 cm^2^ to 1242 cm^2^ in Pyrethroid-PY, 355 cm^2^ to 1325 cm^2^ in Chlorfenapyr-PY LLIN, 526 cm^2^ to 1301 cm^2^ in Pyriproxyfen-PY LLIN and 990 cm^2^ to 2060 cm^2^ in PBO-PY LLIN between t12 and t36 (Additional file 3). There were no significant differences in mean HSA between PY-LLIN, Chlorfenapyr-PY LLIN, and Pyriproxyfen-PY LLIN at any survey time point, while PBO-PY LLIN had substantially higher HSA at each time point than any other study net (Additional file 3). The overall percentage of too—torn nets in the cross-sectional surveys increased from 17% (189/1110) at t12 to 35% (372/1055) at t24 and stabilised between 44% (311/700) at t30 and 43% (432/575) at t36 (Additional file 2). In all net brands, the lower part of the nets (bottom zone) was more damaged than the rest of the zones (Additional file 4). All new nets had similar dimensions (height: 180 cm); however, after 3 years of field use, the height decreased disproportionately between net brands to 170 cm for PY-LLIN, 174 cm for Chlorfenapyr-PY LLIN, 157 cm for Pyriproxyfen-PY LLIN, and 158 cm for PBO-PY LLIN (Additional file 5).

There was no significant association between malaria prevalence and net condition: damaged (OR 0.98, 95% CI 0.71–1.37, p-value = 0.655) and too-torn (OR: 1.07, 95% CI 0.77–1.47, p-value = 0.694) compared to good nets (Table 2; Additional file 10). Malaria infection was significantly lower for children living in clusters that received Chlorfenapyr-PY LLINs compared to those living in clusters that received PY LLINs regardless of the physical condition (Table 2). Children from houses with more than 50% of the sleeping spaces covered by the study nets had lower odds of malaria (OR 0.61, 95% CI 0.42–0.87, p-value = 0.006) than households with fewer sleeping spaces covered. The odds of malaria in all arms increased with time since the net distribution, however, was lower at t36 compared to t24, and this was likely related to the distribution of PBO-PY LLIN in all arms in October 2021 (4 months before t36 survey) in the study area via the school-net programme (SNP). Moreover, children sleeping under nets had lower odds of malaria infection than children not using any nets (Additional file 7). In the surveyed households, the majority of households reported closing the main doors at night, getting inside houses, and sleeping time between 21 and 22 h (Additional file 9).

**Table 2:** Association between net physical condition and malaria prevalence in children aged 6 months to 14 years in cross-sectional surveys.^*^Household study net coverage: is the proportion of sleeping spaces in the household used last night covered by study net; There was no evidence of interaction between net physical condition and net type,  = 0.9814. There was no evidence of interaction between net type and survey timepoint,  = 0.5010

Covariate	%Infection (n/N)	Adjusted OR		95% CI	p-value
Net condition					
Good	24.6 (180/733)	1 (Ref)			
Damaged	25.4 (109/429)	0.98	0.71–1.37		0.655
Too-torn	28.3 (146/516)	1.07	0.77–1.47		0.694
Study arm					
Pyrethroid (PY) LLIN	33.7 (168/498)	1 (Ref)			
Chlorfenapyr-PY LLIN	17.7 (90/508)	0.40	0.23–0.69		0.001
Pyriproxyfen-PY LLIN	28.4 (101/356)	0.80	0.47–1.38		0.430
PBO-PY LLIN	24.1 (76/316)	0.62	0.35–1.08		0.091
Cross-sectional survey					
12 months post-intervention	17.0 (103/607)	1 (Ref)			
24 months post-intervention	36.1 (210/582)	2.93	2.15–3.99		< 0.001
36 months post-intervention	25.0 (122/489)	1.72	1.22–2.43		0.002
Children age					
0–4 years	14.8 (97/656)	1 (Ref)			
5–10 years	30.5 (213/699)	2.86	2.13–3.85		< 0.001
11–14 years	38.7 (125/323)	4.62	3.25–6.58		< 0.001
SES					
Lowest	27.5 (156/567)	1 (Ref)			
Middle	27.2 (155/571)	0.92	0.68–1.26		0.622
Highest	23.0 (124/540)	0.66	0.46–0.93		0.018
Eaves					
Yes	30.7 (167/544)	1 (Ref)			
No	23.6 (268/1134)	0.78	0.59–1.04		0.096
Household study net coverage*					
Too few (< 50%)	33.5 (218)	1 (Ref)			
Moderate/high (> = 50%)	24.9 (1460)	0.61	0.42–0.87		0.006

^*^Household study net coverage: is the proportion of sleeping spaces in the household used last night covered by study net; there was no evidence of interaction between net physical condition and net type,  = 0.9814. There was no evidence of interaction between net type and survey timepoint,  = 0.5010

In the cohort, 5019 children (2256 in year 1 and 2763 in year 2) were assessed alongside the nets they used at the last cohort visit. Of these children, 2239 (99%) in year 1 and 2403 (87%) in year 2 declared to own study nets. These nets were brought for physical condition assessment. Overall, the mean HSA in the cohort nets was 786 cm^2^ for year 1 and 1047 cm^2^ for year 2. Over the two years of cohort study, the overall percentage of too-torn nets increased from 19% (430/2234) at t12 to 33% (801/2397) at t24 (Additional file 2).

There were increased rates of malaria cases in children sleeping under too-torn nets (IRR 1.33 [95% CI 1.12–1.57], p = 0.001) compared to sleeping under good nets (Table 3). Lower rates of malaria cases were associated with living in houses with the highest social-economic status (IRR 0.77 [95% CI 0.63–0.96], p = 0.019), using chlorfenapyr-PY LLIN (IRR 0.49 [95% CI 0.31–0.79], p = 0.004). Higher rates were associated with using study nets that were 2 years old (IRR 1.41 [95% CI 1.19–1.67], p < 0.000). Older children and living in a house with open eaves were not associated with a higher incidence of malaria.

**Table 3:** Association between net physical condition and malaria case incidence in children aged 6 months to 10 years in cohort study

Covariate	Number of clinical episodes	Follow-up time child years	Incidence per child per year	Adjusted rate ratio		95% CI	*p* value
Study net condition							
Good	573	2268.4	0.25	1 (ref)			
Damaged	258	902.9	0.29	1.18		0.97–1.43	0.094
Too-torn	379	1184.7	0.32	1.33		1.12–1.57	0.001
Study arm							
Pyrethroid (PY) LLIN	392	1114.7	0.35	1 (ref)			
Chlorfenapyr-PY LLIN	174	1120.4	0.16	0.49		0.31–0.79	0.004
Pyriproxyfen-PY LLIN	363	1124.4	0.32	1.00		0.64–1.58	0.984
PBO-PY LLIN	281	996.5	0.28	0.83		0.52–1.32	0.433
Cohort year							
Year1	434	1931.4	0.22	1 (ref)			
Year2	776	2424.6	0.32	1.41		1.19–1.67	< 0.001
Children age group							
0–4 years	512	1879.7	0.27	1 (ref)			
5–10 years	698	2476.3	0.28	1.08		0.92–1.27	0.360
Socio-economic status							
Lowest	296	899.6	0.33	1 (ref)			
Middle	273	897.2	0.30	0.96		0.79–1.16	0.654
Highest	217	874.0	0.25	0.77		0.63–0.96	0.019
Eaves							
No	829	3061.1	0.27	1 (ref)			
Yes	381	1294.8	0.29	1.00		0.82–1.21	0.977

In order to assess if the dual-AI LLINs were superior to PY LLINs against incidence regardless of their textile conditions, each net type and condition was compared to a PY LLIN in good condition. During the first year of follow-up, the protective effect of too-torn dual-AI LLINs compared to good PY LLIN against malaria case incidence was strongest for too-torn PBO-PY LLIN (IRR 0.37 [95% CI 0.19–0.72], p = 0.003), borderline for chlorfenapyr-PY LLIN (IRR: 0.45 [95% CI 0.33–0.97], p = 0.053) and no additional protection was given by pyriproxyfen-PY LLIN (IRR 1.15 [95% CI 0.61–2.17], p = 0.660). Sleeping under a good PBO-PY LLIN or a good chlorfenapyr-PY LLIN was more protective than sleeping under a good PY LLIN against malaria case incidence. For children using pyriproxyfen-PY LLIN, however, there was a slight decrease in malaria incidence in children sleeping under damaged nets (0.26 cases per child/year) or good nets (0.24 cases per child/year); however, those differences were not significant compared to good PY LLIN (IRR = 0.74 [95% 0.38–1.46], p = 0.386) for damaged pyriproxyfen-PY LLIN and (IRR = 0.78 [95% 0.46–1.34], p = 0.369) for good pyriproxyfen-PY LLIN (Table 4).

**Table 4:** Association between net physical condition and malaria case incidence in children aged 6 months to 10 years in year 1 in cohort study

LLIN type and condition	Number of clinical episodes	Follow-up time child years	Incidence per child per year	Adjusted rate ratio	95% CI	p value
Pyrethroid (PY) LLIN						
Good	104	313.48	0.33	1		
Damaged	36	95.67	0.38	0.90	0.57–1.43	0.654
Too-torn	30	81.17	0.37	0.93	0.56–1.54	0.778
Pyriproxyfen-PY LLIN						
Good	83	342.67	0.24	0.78	0.46–1.34	0.369
Damaged	22	84.64	0.26	0.74	0.38–1.46	0.386
Too-torn	34	84.41	0.40	1.15	0.61–2.17	0.660
PBO-PY LLIN						
Good	33	199.70	0.17	0.43	0.23–0.80	0.0076
Damaged	12	70.58	0.17	0.61	0.28–1.33	0.2128
Too-torn	22	174.74	0.13	0.37	0.19–0.72	0.0033
Chlorfenapyr-PY LLIN						
Good	34	309.56	0.11	0.35	0.28–0.71	0.001
Damaged	15	96.40	0.16	0.51	0.38–1.03	0.068
Too-torn	10	79.30	0.13	0.45	0.33–0.97	0.053

In year 2, compared to those sleeping under good PY-LLIN, only children sleeping under chlorfenapyr-PY LLIN in good condition had a significant and more substantial protective effect (IRR = 0.49 [95% CI 0.52–1.37], p = 0.006) against malaria case incidence (Table 5). There was no reduced risk of infection associated with sleeping under too-torn PBO-PY LLIN (IRR = 1.36 [95% 0.86–2.16], p = 0.186) and too-torn Pyriproxyfen-PY LLIN (IRR = 1.46 [95% CI 0.89–2.37], p = 0.131) in year 2 compared to PY-LLIN in good condition.

**Table 5:** Association between net physical condition and malaria case incidence in children aged 6 months to 10 years in year 2 in cohort study

LLIN type and condition	Number of clinical episodes	Follow-up time child years	Incidence per child per year	Rate ratio	95% CI	p value
Pyrethroid (PY) LLIN						
Good	107	312.18	0.34	1		
Damaged	62	159.22	0.39	1.19	0.83–1.70	0.353
Too-torn	53	152.95	0.35	1.07	0.74–1.56	0.713
Pyriproxyfen-PY LLIN						
Good	80	299.81	0.27	0.85	0.52–1.37	0.495
Damaged	57	133.47	0.43	1.37	0.82–2.28	0.231
Too-torn	87	179.46	0.48	1.46	0.89–2.37	0.131
PBO-PY LLIN						
Good	67	181.21	0.37	1.18	0.72–1.94	0.514
Damaged	26	91.19	0.29	0.98	0.54–1.77	0.951
Too-torn	119	275.63	0.43	1.36	0.86–2.16	0.186
Chlorfenapyr-PY LLIN						
Good	50	317.19	0.16	0.49	0.29–0.81	0.006
Damaged	36	169.21	0.21	0.67	0.39–1.16	0.150
Too-torn	30	153.87	0.19	0.64	0.36–1.14	0.129

## Discussion

As part of a cluster randomised trial of dual-active ingredient malaria vector control interventions, the textile conditions of the dual-AI LLINs was assessed [namely: chlorfenapyr-PY LLIN, pyriproxyfen-PY LLIN, and a PBO-PY LLIN] after 3 years of use in the community [8]. The associations between net conditions and malaria prevalence (from repeated cross-sectional surveys) and incidence (from a cohort study) in Mwanza region, Tanzania were explored.

In this study, based on the cross-sectional survey data, there was no evidence indicating that the condition of the net was associated with malaria prevalence. Indeed, it was observed that good, damaged, and too-torn study nets appeared to offer similar levels of personal protection against malaria infection prevalence after adjusting for several covariates, such as net age, child age, presence of eaves in the house, socio-economic status, and household level net coverage. The strong protection provided by high household coverage of nets (> 50%) highlights the importance of promoting high levels of ownership and retention of nets in all households. Additionally, from the repeated cross-sectional measures, also it was observed that study nets offered protection to those sleeping under them against malaria infection, and that sleeping under any net with holes provided more protection than sleeping without a net at all, similar to studies undertaken in Malawi [17, 18, 23], and Equatorial Guinea [18].

LLINs, as the principal form of malaria vector control in the study area, reduced malaria infection by 54%, regardless of the net type and whether they had holes, compared to not using a net at all. Notably, even users of pyrethroid-only LLINs were far more protected than non-net users. This finding supports existing evidence that pyrethroid-only LLINs still provide protection compared to sleeping without a net, even in insecticide-resistant settings [17, 23, 24]. Several studies have reported on the association between increased levels of damage of pyrethroid-only nets and increased malaria infection [18, 25] while others reported no association [26, 27]. However, while no impact of differences in net integrity on malaria prevalence was seen, in the cohort study, there was a strong association between net textile condition and malaria case incidence, unlike the cohort study in Malawi [23]. In the first year, when the insecticides were in suitable [8] concentrations, torn chlorfenapyr-PY LLIN and PBO-PY LLIN were better than good PY-LLIN. However, in the second year, only good chlorfenapyr-PY LLIN was better than good PY-LLIN; this may be explained by waning insecticide concentrations in the dual-AI LLINs over time [8].

Torn pyriproxyfen-PY LLINs (in the first and second year) and torn PBO-PY LLINs (during the second year) of use did not provide superior protection against malaria case incidence compared to good PY LLINs. This is consistent with results generated recently in Tanzania [8] and Benin [9], where pyriproxyfen-PY LLINs and PBO-PY LLIN did not perform well during the second year. This study supports and adds weight to previous studies that suggested the impaired effectiveness of these two products is likely related to poor fabric integrity, leading to an unexpected decline in community coverage. However, a systematic review of 22 published studies reported that wear and tear were not identified as a reason for not using mosquito nets when they are the only nets available [28].

In this study, ownership, usage, and textile conditions of all nets declined swiftly over the 3 years after net distribution. The decline was more marked in the PBO-PY LLIN arm followed by pyriproxyfen-PY LLINs, consistent with the results generated recently in Tanzania in a prospective net cohort study done with the same net [29]. It is fairly standard to see a reduction in usage and ownership in places with no continuous distribution of nets [18]. However, through the continuous distribution of nets in antenatal care (ANC) clinics and the expanded programme on immunization (EPI) and school-based programmes coverage of other nets in the study area was kept high [30].

In all nets, the damage was most severe in the bottom part [12, 31]. This part has been reported to have limited contribution to mosquito prevention [31] as it is recommended to tuck the net under the mat or mattress. However, improper tucking of the bed net is not uncommon practice [32–34], and net tucking is very challenging without a proper bed frame; in this study, more than half of the sleeping spaces in surveyed households had no bed frames. For untucked or partially tucked nets, the bottom part will still allow mosquitoes to penetrate. Enhancing the bottom part might remedy nets from early hole development and further enlargement, although other studies have shown that dense knitting in this part of the net did not necessarily make it more durable [35].

Similarities in physical properties (fibers, denier, and integrity) between some nets [chlorfenapyr-PY LLIN and PY-LLIN], but differences in protective efficacy against malaria infection emphasize the pivotal role of non-pyrethroid insecticide and synergists (chlorfenapyr and PBO [36]) in malaria vector control. In addition, this study demonstrates that the resilient physical integrity of the nets on its own is not enough to provide sustained protection to users of nets in good conditions against bites of malaria-transmitting mosquitoes even in settings of high net coverage, as it was in the cohort children where > 80% used nets. This is contrary to the review by Okumu (2020) [37], which suggested that physically durable nets could serve a similar purpose as insecticidal nets.

### Limitations

In this study, the net condition at the end of the year against cumulative incidence over the year was assessed; likely, the nets were not in that condition for the whole year. However, when the association between malaria prevalence and net condition in cohort children during their last visits was assessed, it showed similar results. The number of study nets to be assessed decreased over time due decline in coverage, leading to a small number of cross-sectional nets assessed towards the end. However, a better and direct comparison of the protective effect of nets was attained with the cohort nets, which had high access and usage across the study. Net users in the cross-sectional survey tend to replace lightly damaged nets with readily available new ones, likely, the majority of the nets evaluated towards the end of the study were still new or put into usage a few months previously. Finally, there could be a possibility of cohort children not using the same net all the time. However, to reduce the potential impact of children using other nets, the community health worker (CHW) visited the cohort children to insist, and monitor appropriate net usage.

## Conclusion

These results provide evidence that sleeping under too-torn chlorfenapyr-PY LLIN and PBO-PY LLIN offered superior protection compared to sleeping under good standard net, and, the protective effect of PBO-PY LLIN diminishes as PBO-treated nets age. The results also show that, when assessing net integrity independent of the protective effect against malaria, chlorfenapyr-PY LLIN (Interceptor G2, polyester net) nets are physically more durable than PBO-PY LLIN (Olyset Plus, polyethylene net) but relatively similar to pyriproxyfen-PY LLIN (Royal Guard, polyethylene net). The future strategies for control programmes reliant on the dual-AI LLINs, therefore, not only seek to provide new nets to households that do not have nets but also instigate strategies to inform the population that even a torn dual-AI LLIN is better than an intact standard net or sleeping without a net; small holes in nets should not motivate households to discard the nets. Furthermore, there should be an emphasis on proper net care and repair practices among users, as well as the necessity for manufacturers to develop physically durable LLINs.

### Supplementary Information

**Additional file1:** Characteristics of the study nets distributed as part of the project in January 2019

**Additional file2:** Study nets in good, damaged and too-torn condition (data from cross-sectional and cohort nets)

**Additional file3:** Multiple comparisons of the mean surface area of the holes in the study nets between nets types and age

**Additional file4:** Mean holes per study net per survey by zones (data from cross-sectional survey)

**Additional file5:** Mean height of the study net at t0, t30 and t36

**Additional file6:** Source of nets in the study area

**Additional file7:** Association between sleeping under of different net physical condition and malaria prevalence in children aged 6 months to 14 years

**Additional file8:** Association between net physical condition and malaria prevalence in children aged 6 months to 10 years

**Additional file9:** Time people get inside the house and close the main doors

**Additional file10:** Interaction between net physical condition and net type

## Data Availability

The datasets generated and/or analysed during the current study are not publicly available due strict laws in Tanzania that restrict data to be shared outside the country without a Data Transfer agreement (DTA.pdf (nimr.or.tz)). For the interested researchers, the DTA can be completed by the help of Mr Eliud Lukole (ellylufi@ymail.com) or Prof. Natacha Protopopoff (natacha.protopopoff@lshtm.ac.uk).

## Abbreviations

LLIN: Long-lasting insecticidal net
AI: Active ingredient
PY: Pyrethroid
PBO: Piperonyl butoxide
PPF: Pyriproxyfen
RCT: Randomized controlled trial
IRR: Incidence rate ratio
SNP: School net programme
