# Supplementary material for: Will a lack of fabric durability be their downfall? Impact of textile durability on the efficacy of three types of dual-active-ingredient long-lasting insecticidal nets: a secondary analysis on malaria prevalence and incidence from a cluster-randomized trial in north-west Tanzania
Source: Malar J. 2024 Jun 28;23:199. doi: 10.1186/s12936-024-05020-y (PMC11212245; doi:10.1186/s12936-024-05020-y)
Supplement: Supplementary file 1 — Additional file1: Characteristics of the study nets distributed as part of the project in January 2019 [file 12936_2024_5020_MOESM1_ESM.docx]

Appendix 1: Characteristics of the study nets distributed as part of the project in January 2029

| **LLIN brand** | **Dose AI/m2 (gAI/kg) of netting fabric)** | **Physical properties** | | | | | |
| --- | --- | --- | --- | --- | --- | --- | --- |
|  |  | **Fibre** | **Denier** | **Bursting strength** | **Dimensional stability*** | **Netting mesh size** | **Net dimensions** |
| Interceptor® | Alpha-cypermethrin 200 mg | Polyester | 100 | ≥405 kPa | ≤5% | 24 holes/cm² | Length: 180 cm Width:160cm Height: 180cm |
| Interceptor® G2 | Alpha-cypermethrin 100 mg | Polyester | 100 | ≥405 kPa | ≤5% | 24 holes/cm² |  |
|  | + Chlorfenapyr 200 mg |  |  |  |  |  |  |
| Royal guard® | Alpha-cypermethrin 220 mg | Polyethylene | 120 | ≥400 kPa | ≤5% | 20 holes/cm² |  |
|  | + Pyriproxyfen 220 mg |  |  |  |  |  |  |
| Olyset® Plus | Permethrin 800 mg + PBO 400 mg | Polyethylene | 150 | ≥250 kPa | ≤5% | 6 holes/cm² |  |
| **Dimensional stability of netting to washing (shrinkage/expansion in both directions)* | | | | | | | |
