# Supplementary material for: Will a lack of fabric durability be their downfall? Impact of textile durability on the efficacy of three types of dual-active-ingredient long-lasting insecticidal nets: a secondary analysis on malaria prevalence and incidence from a cluster-randomized trial in north-west Tanzania
Source: Malar J. 2024 Jun 28;23:199. doi: 10.1186/s12936-024-05020-y (PMC11212245; doi:10.1186/s12936-024-05020-y)
Supplement: Supplementary file 3 — Additional file3: Multiple comparisons of the mean surface area of the holes in the study nets between nets types and age [file 12936_2024_5020_MOESM3_ESM.docx]

Appendix 3: Multiple comparisons of the mean surface area of the holes in the study nets between nets types and age

| **Hole surface area (survey: net type)** | **Mean** |  | **Std. Err.** | **Bonferroni Groups*** | **95% CI** |  |
| --- | --- | --- | --- | --- | --- | --- |
| 12months: Pyrethroid (PY)-LLIN | 340 |  | 111.72 | A | 221.82-458.61 |  |
| 12months: Chlorfenapyr-PY LLIN | 355 |  | 102.14 | A | 237.02-473.22 |  |
| 12months: Pyriproxyfen-PY LLIN | 527 |  | 121.49 | AB | 300.27-752.85 |  |
| 24months: Pyrethroid (PY)-LLIN | 755 |  | 108 | ABC | 578.19-932.05 |  |
| 12months: PBO-PY LLIN | 990 |  | 120.99 | BCD | 742.84-1238.14 |  |
| 24months: Chlorfenapyr-PY LLIN | 994 |  | 111.53 | BCD | 784.35-1203.8 |  |
| 30months: Pyrethroid (PY)-LLIN | 1039 |  | 126.59 | BCD | 818.87-1259.36 |  |
| 24months: Pyriproxyfen-PY LLIN | 1114 |  | 113.12 | CD | 872.47-1355.44 |  |
| 36months: Pyrethroid (PY)-LLIN | 1242 |  | 104.46 | CD | 918.67-1564.86 |  |
| 36months: Pyriproxyfen-PY LLIN | 1301 |  | 133.37 | CDE | 1004.88-1597.98 |  |
| 30months: Chlorfenapyr-PY LLIN | 1358 |  | 133.7 | CDEF | 1122.7-1592.31 |  |
| 36months: Chlorfenapyr-PY LLIN | 1325 |  | 101.4 | DE | 1007.21-1642.58 |  |
| 24months: PBO-PY LLIN | 1621 |  | 138.29 | DEF | 1116.51-2126.07 |  |
| 30months: Pyriproxyfen-PY LLIN | 1513 |  | 147.73 | DEF | 994.3-2031.84 |  |
| 30months: PBO-PY LLIN | 2000 |  | 175.12 | EF | 1507.85-2492.16 |  |
| 36months: PBO-PY LLIN | 2060 |  | 162.33 | F | 1507.07-2612.17 |  |
| *Means sharing a letter in the Bonferroni Groups label are not significantly different at the 5% level. | | | | | |  |
