# Supplementary material for: Will a lack of fabric durability be their downfall? Impact of textile durability on the efficacy of three types of dual-active-ingredient long-lasting insecticidal nets: a secondary analysis on malaria prevalence and incidence from a cluster-randomized trial in north-west Tanzania
Source: Malar J. 2024 Jun 28;23:199. doi: 10.1186/s12936-024-05020-y (PMC11212245; doi:10.1186/s12936-024-05020-y)
Supplement: Supplementary file 5 — Additional file5: Mean height of the study net at t0, t30 and t36 [file 12936_2024_5020_MOESM5_ESM.docx]

Appendix 5: Mean height of the study net at t0, t30 and t36
