# Supplementary material for: Will a lack of fabric durability be their downfall? Impact of textile durability on the efficacy of three types of dual-active-ingredient long-lasting insecticidal nets: a secondary analysis on malaria prevalence and incidence from a cluster-randomized trial in north-west Tanzania
Source: Malar J. 2024 Jun 28;23:199. doi: 10.1186/s12936-024-05020-y (PMC11212245; doi:10.1186/s12936-024-05020-y)
Supplement: Supplementary file 6 — Additional file6: Source of nets in the study area [file 12936_2024_5020_MOESM6_ESM.docx]

Appendix 6: Source of nets in the study area

| **Variable** | **12 months** | **24 months** | **30 months** | **36 months** |
| --- | --- | --- | --- | --- |
| % Trial nets distributed in January 2019 (n) | 63.99 (1926) | 49.46 (1243) | 44.98 (775) | 34.39 (1122) |
| % School Net Program (n) | 9.7 (292) | 8.4 (211) | 12.13 (209) | 27.28 (890) |
| % Antenatal clinic/immunization program (n) | 15.51 (467) | 29.17 (733) | 35.4 (610) | 28.65 (935) |
| % 2015 Universal Campaign (n) | 1.1 (33) | 1.31 (33) | 0.7 (12) | 0.34 (11) |
| % Other faith-based programs (n) | 2.29 (69) | 8.24 (207) | 2.15 (37) | 4.72 (154) |
| % Purchased full price (n) | 0.86 (26) | 1.39 (35) | 2.96 (51) | 2.57 (84) |
| % Received for free by relatives (n) | 0.5 (15) | 0.96 (24) | 0.52 (9) | 0.95 (31) |
| % Don’t know the source (n) | 6.05 (182) | 1.07 (27) | 1.16 (20) | 1.1 (36) |
