# Supplementary material for: Will a lack of fabric durability be their downfall? Impact of textile durability on the efficacy of three types of dual-active-ingredient long-lasting insecticidal nets: a secondary analysis on malaria prevalence and incidence from a cluster-randomized trial in north-west Tanzania
Source: Malar J. 2024 Jun 28;23:199. doi: 10.1186/s12936-024-05020-y (PMC11212245; doi:10.1186/s12936-024-05020-y)
Supplement: Supplementary file 7 — Additional file7: Association between sleeping under of different net physical condition and malaria prevalence in children aged 6 months to 14 years [file 12936_2024_5020_MOESM7_ESM.docx]

Appendix 7: **Association between sleeping under of different net physical condition and malaria prevalence in children aged 6 months to 14 years in cross-sectional surveys**

|  | **% infected (N)** |  | **Univariable analysis** | | |  | **Multivariable analysis** | | |
| --- | --- | --- | --- | --- | --- | --- | --- | --- | --- |
| **Covariate** |  |  | **Crude OR** | **95% CI** | **p-value** |  | **Adjusted OR** | **95% CI** | **p-value** |
| **Condition of net** |  |  |  |  |  |  |  |  |  |
| Not using any net | 46.1 (993) |  | 1 (Ref) |  |  |  | 1 (Ref) |  |  |
| Too-torn study LLINs | 28.3 (516) |  | 0.47 | 0.37-0.61 | <0.001 |  | 0.60 | 0.45-0.80 | 0.001 |
| Damaged study LLINs | 25.4 (429) |  | 0.40 | 0.31-0.53 | <0.001 |  | 0.56 | 0.41-0.77 | <0.001 |
| Good study LLINs | 24.6 (733) |  | 0.33 | 0.26-0.42 | <0.001 |  | 0.56 | 0.42-0.75 | <0.001 |
| **Study arm** |  |  |  |  |  |  |  |  |  |
| Pyrethroid (PY) LLIN | 40.9 (729) |  | 1 (Ref) |  |  |  | 1 (Ref) |  |  |
| Chlorfenapyr-PY LLIN | 23.8 (748) |  | 0.47 | 0.27-0.79 | 0.005 |  | 0.40 | 0.24-0.69 | 0.001 |
| Pyriproxyfen-PY LLIN | 35.3 (632) |  | 0.78 | 0.45-1.30 | 0.318 |  | 0.65 | 0.38-1.12 | 0.117 |
| PBO-PY LLIN | 34.5 (562) |  | 0.76 | 0.46-1.32 | 0.352 |  | 0.68 | 0.40-1.17 | 0.161 |
| **Cross-sectional survey** |  |  |  |  |  |  |  |  |  |
| 12 months post-intervention | 20.8 (816) |  | 1 (Ref) |  |  |  | 1 (Ref) |  |  |
| 24 months post-intervention | 41.4 (865) |  | 2.93 | 2.32-3.70 | <0.001 |  | 2.92 | 2.27-3.75 | <0.001 |
| 36 months post-intervention | 36.9 (990) |  | 2.38 | 1.89-2.99 | <0.001 |  | 1.81 | 1.40-2.34 | <0.001 |
| **Children age** |  |  |  |  |  |  |  |  |  |
| 0-4 years | 17.0 (870) |  | 1 (Ref) |  |  |  | 1 (Ref) |  |  |
| 5-10 years | 38.8 (1174) |  | 3.26 | 2.60-4.10 | <0.001 |  | 3.27 | 2.57-4.15 | <0.001 |
| 11-14 years | 46.1 (627) |  | 4.67 | 3.62-6.03 | <0.001 |  | 4.70 | 3.58-6.17 | <0.001 |
| **Socio-economic status** |  |  |  |  |  |  |  |  |  |
| Lowest | 35.5 (901) |  | 1 (Ref) |  |  |  | 1 (Ref) |  |  |
| Middle | 35.2 (887) |  | 1.01 | 0.82-1.26 | 0.892 |  | 0.97 | 0.76-1.23 | 0.773 |
| Highest | 29.6 (883) |  | 0.79 | 0.63-0.98 | 0.034 |  | 0.67 | 0.51-0.88 | 0.004 |
| **Eaves** |  |  |  |  |  |  |  |  |  |
| Yes | 40.0 (893) |  | 1 (Ref) |  |  |  | 1 (Ref) |  |  |
| Not | 30.2 (1778) |  | 0.65 | 0.54-0.79 | <0.001 |  | 0.72 | 0.58-0.90 | 0.005 |
| **Household study net coverage** | |  |  |  |  |  |  |  |  |
| Too few (<=50%) | 45.6 (800) |  | 1 (Ref) |  |  |  | 1 (Ref) |  |  |
| Moderate/high (>50%) | 27.4 (1775) |  | 0.46 | 0.38-0.55 | <0.001 |  | 0.69 | 0.55-0.88 | 0.003 |
| *Household study net coverage= proportion of sleeping spaces in the household used last night covered by study net | | | | | | | | | |
