# Supplementary material for: Will a lack of fabric durability be their downfall? Impact of textile durability on the efficacy of three types of dual-active-ingredient long-lasting insecticidal nets: a secondary analysis on malaria prevalence and incidence from a cluster-randomized trial in north-west Tanzania
Source: Malar J. 2024 Jun 28;23:199. doi: 10.1186/s12936-024-05020-y (PMC11212245; doi:10.1186/s12936-024-05020-y)
Supplement: Supplementary file 8 — Additional file8: Association between net physical condition and malaria prevalence in children aged 6 months to 10 years [file 12936_2024_5020_MOESM8_ESM.docx]

Appendix 8: **Association between net physical condition and malaria prevalence (in children cohort survey) in children aged 6 months to 10 years at the end of follow-up period**

| **Condition of the net** | **Odds Ratio** | | **95%CI** | | **p-values** | |  |
| --- | --- | --- | --- | --- | --- | --- | --- |
| Good | | 1 (Ref) | |  | |  | |
| Damaged | | 1.09 | | 0.86-1.38 | | 0.469 | |
| Too-torn | | 1.40 | | 1.13-1.73 | | 0.002 | |
| **Net type** | |  | |  | |  | |
| Pyrethroid (PY)-LLIN | | 1 (Ref) | |  | |  | |
| Chlorfenapyr-PY LLIN | | 0.83 | | 0.46-1.52 | | 0.553 | |
| Pyriproxyfen-PY LLIN | | 0.32 | | 0.17-0.59 | | <0.001 | |
| PBO-PY LLIN | | 0.63 | | 0.35-1.15 | | 0.134 | |
| **Cohort year** | |  | |  | |  | |
| End of year1 | | 1 (Ref) | |  | |  | |
| End of year2 | | 6.38 | | 5.15-7.91 | | <0.001 | |
| **Age group** | |  | |  | |  | |
| 0-4yrs | | 1 (Ref) | |  | |  | |
| 5-10yrs | | 2.22 | | 1.84-2.69 | | <0.001 | |
