# Supplementary material for: Will a lack of fabric durability be their downfall? Impact of textile durability on the efficacy of three types of dual-active-ingredient long-lasting insecticidal nets: a secondary analysis on malaria prevalence and incidence from a cluster-randomized trial in north-west Tanzania
Source: Malar J. 2024 Jun 28;23:199. doi: 10.1186/s12936-024-05020-y (PMC11212245; doi:10.1186/s12936-024-05020-y)
Supplement: Supplementary file 9 — Additional file9: Time people get inside the house and close the main doors [file 12936_2024_5020_MOESM9_ESM.docx]

Appendix 9: Time people get inside the house and close the main doors

| **time went to sleep** | Chlorfenapyr-PY LLIN | PBO-PY LLIN | Pyriproxyfen-PY LLIN | Pyrethroid (PY) LLIN |
| --- | --- | --- | --- | --- |
|  |  |  |  |  |
| % before21hrs (n) | 28.82 (441) | 27.73 (353) | 28.99 (409) | 27.06 (417) |
|  |  |  |  |  |
| % after21hrs (n) | 71.18 (1089) | 72.27 (920) | 71.01 (1002) | 72.94 (1124) |
