# Supplementary material for: Will a lack of fabric durability be their downfall? Impact of textile durability on the efficacy of three types of dual-active-ingredient long-lasting insecticidal nets: a secondary analysis on malaria prevalence and incidence from a cluster-randomized trial in north-west Tanzania
Source: Malar J. 2024 Jun 28;23:199. doi: 10.1186/s12936-024-05020-y (PMC11212245; doi:10.1186/s12936-024-05020-y)
Supplement: Supplementary file 10 — Additional file10: Interaction between net physical condition and net type [file 12936_2024_5020_MOESM10_ESM.docx]

Appendix 10: Interaction between net physical condition and net type

| **Covariate** | | **% infected (n/N)** | |  | | **Adjusted OR** | **95% CI** | | **p-value** | | | |
| --- | --- | --- | --- | --- | --- | --- | --- | --- | --- | --- | --- | --- |
| **Interaction between net condition and net type** | | |  | |  | | |  | |  |  |  |
| Good: PY-LLIN | 34.05 (79/232) | |  | | 1 (Ref) | | |  | |  |  |  |
| Damaged: PY-LLIN | 31.82 (42/132) | |  | | 1.01 | | | 0.58-1.77 | | 0.963 | |  |
| Too-torn: PY-LLIN | 35.07 (47/134) | |  | | 0.99 | | | 0.57-1.73 | | 0.975 |  |  |
| Good: Pyriproxyfen-PY LLIN | 23.13 (34/147) | |  | | 0.63 | | | 0.32-1,27 | | 0.200 |  |  |
| Damaged: Pyriproxyfen-PY LLIN | 30.43 (28/92) | |  | | 0.77 | | | 0.37-1.62 | | 0.496 |  |  |
| Too-torn: Pyriproxyfen-PY LLIN | 33.33 (39/117) | |  | | 0.86 | | | 0.43-1.73 | | 0.676 |  |  |
| Good: PBO-PY LLIN | 23.81 (30/126) | |  | | 0.70 | | | 0.35-1.42 | | 0.324 |  |  |
| Damaged: PBO-PY LLIN | 21.92 (16/73) | |  | | 0.52 | | | 0.23-1.19 | | 0.121 |  |  |
| Too-torn: PBO-PY LLIN | 25.64 (30/117) | |  | | 0.64 | | | 0.31-1.31 | | 0.223 |  |  |
| Good: Chlorfenapyr-PY LLIN | 16.23 (37/228) | |  | | 0.45 | | | 0.23-0.87 | | 0.017 |  |  |
| Damaged: Chlorfenapyr-PY LLIN | 17.42 (23/132) | |  | | 0.43 | | | 0.20-0.89 | | 0.023 |  |  |
| Too-torn: Chlorfenapyr-PY LLIN | 20.27 (30/148) | |  | | 0.47 | | | 0.23-0.96 | | 0.040 |  |  |
